# Bacteriocins: Potential for Human Health

**DOI:** 10.1155/2021/5518825

**Published:** 2021-04-10

**Authors:** Fuqing Huang, Kunling Teng, Yayong Liu, Yanhong Cao, Tianwei Wang, Cui Ma, Jie Zhang, Jin Zhong

**Affiliations:** ^1^State Key Laboratory of Microbial Resources, Institute of Microbiology, Chinese Academy of Sciences, Beijing 100101, China; ^2^University of Chinese Academy of Sciences, Beijing 100008, China; ^3^The Animal Husbandry Research Institute of Guangxi Zhuang Autonomous Region, Nanning 530000, China

## Abstract

Due to the challenges of antibiotic resistance to global health, bacteriocins as antimicrobial compounds have received more and more attention. Bacteriocins are biosynthesized by various microbes and are predominantly used as food preservatives to control foodborne pathogens. Now, increasing researches have focused on bacteriocins as potential clinical antimicrobials or immune-modulating agents to fight against the global threat to human health. Given the broad- or narrow-spectrum antimicrobial activity, bacteriocins have been reported to inhibit a wide range of clinically pathogenic and multidrug-resistant bacteria, thus preventing the infections caused by these bacteria in the human body. Otherwise, some bacteriocins also show anticancer, anti-inflammatory, and immune-modulatory activities. Because of the safety and being not easy to cause drug resistance, some bacteriocins appear to have better efficacy and application prospects than existing therapeutic agents do. In this review, we highlight the potential therapeutic activities of bacteriocins and suggest opportunities for their application.

## 1. Introduction

Many human diseases are associated with bacterial infections. While antibiotics have played an instrumental role in the fight against them, the widespread misuse of antibiotics has led to the emergence of a serious worldwide drug resistance problem; the discovery of new antimicrobial drugs is therefore urgent [1]. Bacteriocins are peptides with antibacterial activity synthesized by bacterial ribosomes, and they are usually inhibitory to proximate bacteria. [2] Bacteriocins are typically classified into Class I (heat-stable posttranslationally modified peptides below 10 kDa) including lanthipeptide, lasso peptide, head-to-tail cyclized peptides, thiopeptide, glycosylated bacteriocin, and sactipeptide; Class II (heat-stable unmodified small peptides below 10 kDa) including IIa/b/c/d; and Class III (thermally unstable peptides larger than 10 kDa) [3]. Due to its unique mechanism of action, such as modification of the pyrophosphate moiety of lipid-II, bacteriocins have a relatively narrower spectrum of inhibition against bacteria and are less likely to develop widespread drug resistance than antibiotics [4, 5].

Bacteriocins can inhibit many disease-causing bacteria, including some antibiotic-resistant strains, suggesting the potential application of bacteriocins in antagonizing pathogenic infections. The human body (e.g., in gastrointestinal tract, respiratory tract, and skin and reproductive tract) has a large number of microorganisms, and the host microbiota is constantly interacting with the host cells. Many human microorganisms can produce bacteriocins which are reported to be closely related to human health, such as promoting the balance of the gut microbiota and inhibiting the invasion of foreign pathogenic bacteria [6]. In addition to inhibiting pathogenic bacteria, bacteriocins have shown inhibitory effects on a wide range of cancer cells as well as modulating effects on inflammation and immunity, suggesting that they also show anticancer and anti-inflammatory activities. Therefore, bacteriocins have a great potential for application in human health.

In recent years, there have been some reports on bacteriocins and human health, but there is still a lack of systematic reviews in this field of research. Therefore, it is necessary to summarize the bacteriocins produced by different bacteria and their beneficial effects on human health, so as to provide a theoretical basis for the research and development of bacteriocins. In this review, we summarized the antibacterial, anticancer, anti-inflammatory, and immune-modulatory activities of bacteriocins and concluded their mechanisms of action.

## 2. Functional Properties and Mechanisms of Bacteriocins

**Figure 1:**
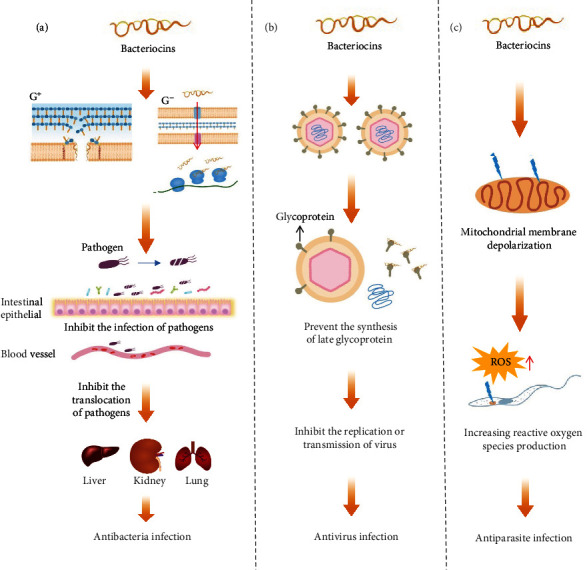
Bacteriocins protect the human body from infection by inhibiting a wide variety of pathogenic microorganisms via different mechanisms. (a) For bacteria, bacteriocins can directly kill pathogenic bacteria by inhibiting the bacteria cell wall biosynthesis by complexing the lipid II and forming the pore in cell membrane, disrupting bacterial population sensing as a signaling molecule, or enters the cell via a transporter and interacts with critical enzymes (e.g., ATP-dependent protease). This eliminates the presence of pathogenic bacteria in the organism and reduces their migration to various extraintestinal organs, i.e., the lung, kidney, and liver. (b) For viruses, bacteriocins can inhibit the proliferation or transfer of viruses by blocking the synthesis of glycoproteins in the late stage of virus replication. (c) For parasites, bacteriocins can inhibit the parasites through mitochondrial membrane depolarization and reactive oxygen species production.

Pathogenic microorganisms pose a major threat to human health and may even endanger human lives. It is predicted that millions of people will die from bacterial infections in the coming decades, because of the emergence of multidrug-resistant (MDR) bacteria [7, 8]. Despite the important contribution of antibiotics in the fight against pathogenic infections, the widespread use and misuse of antibiotics have led to some serious adverse consequences, such as the emergence of superbugs. [9] New compounds for inhibiting the multiresistant pathogens and limiting the spread of antibiotic resistance are urgently needed. Bacteriocins are ribosomally synthesized antimicrobial peptides. Some bacteriocins need to be modified by a posttranslational modified enzyme system and transported by a special transport system to outside of the cell to exert their biological activities (e.g., lantibiotics) [10]. In contrast to antibiotics, the unique mechanism of action (binds the pyrophosphate moiety of Lipid-II [11]) prevents bacteriocins from developing resistance, and treating pathogenic infections with bacteriocins or bacteriocins in combination with antibiotics instead of antibiotics can reduce the overuse of antibiotics, thereby reducing the spread of antibiotic resistance. [5, 12] Moreover, some strains that are resistant to antibiotics appear to have a higher susceptibility to antimicrobial peptides. [13] Leon et al. points out that the mechanism of antimicrobial activities of bacteriocins is completely different from that of antibiotics, which indicated that bacteriocins will be possible as “new age infection fighters” [14]. Some bacteriocins show inhibitory activities against pathogenic microorganisms and can effectively inhibit infections of the human body by pathogenic microorganisms. This suggests that bacteriocin is an effective alternative for the treatment of pathogenic microbial infections. The anti-infection effects of bacteriocins and mechanisms are summarized in Table 1 and Figure 1. Many bacteriocins (e.g., lanthipeptides) demonstrate inhibitory activity against the pathogens. In addition, some bacteriocins have demonstrated inhibitory effects on viruses and parasites.

### 2.1. Inhibiting Bacterial Infections

Many bacteriocins typically exhibit antibacterial activity against the critical pathogenic bacteria, including some antibiotic-resistant Gram-positive (G^+^) bacteria including *Mycobacterium tuberculosis*, methicillin-resistant *Staphylococcus aureus* (MRSA), *Listeria monocytogenes*, vancomycin-resistant enterococci (VRE), *Clostridium difficile*, and Gram-negative (G^−^) bacteria including *Escherichia coli* and *Salmonella enterica*. Bacteriocins exert their antimicrobial action through inhibiting the bacteria cell wall biosynthesis by complexing the lipid II and forming the pore in cell membrane, disrupting bacterial population sensing as a signaling molecule, or targeting the ATP-dependent protease, or binding to a site on 23S rRNA and inhibits elongation factor-dependent reactions (Table 1).

Nisin, produced by *Lactococcus lactis*, is the most researched and developed bacteriocin. Since nisin was found in 1928, it has been used for decades as a safe, natural food biopreservative that significantly inhibits the growth of a wide range of pathogenic microorganisms [15]. For example, nisin can inhibit the growth of *Streptococcus pneumonia* [16] which could cause the disease of pneumonia, meningitis, and sepsis. In addition, nisin has an inhibitory effect on many pathogenic bacteria and ameliorates infections caused by these pathogens, such as respiratory tract infections caused by *S. aureus* [17] and gastrointestinal infections by VRE in mice [18].

In addition, many diseases associated with pathogenic bacterial infections can be treated by bacteriocin interventions. *M*. *tuberculosis* is the pathogen that causes tuberculosis, which affects a quarter of the world's population. Some bacteriocins have been reported to inhibit *M*. *tuberculosis in vitro*. For example, griselimycin, a cyclic bacteriocin, is effective in curing mice infected with tuberculosis *in vivo.* [19] *S. aureus* infections can lead to diseases such as mastitis and bacteraemia. Laterosporulin10, microbisporicin, NVB333, and mersacidin can inhibit *S. aureus in vitro* and/or *in vivo*, thereby treating respiratory tract, foot, abdominal cavity, and nasal cavity of *S. aureus* infection. [20–22] *L. monocytogenes* is the pathogen of listeriosis. It mainly uses food as a vector and is one of the deadliest foodborne pathogens, causing 20 to 30% of the infected deaths [23]. Moreover, it also has the ability to cross the intestinal barrier to reach the blood and extraintestinal organs. Some studies have reported that certain bacteriocins, such as pediocin PA-1, lactocin AL705, and enterocin CRL35, inhibit the growth of *L*. *monocytogenes* and also reduce the number of their passage through the intestinal barrier [24, 25]. *E. coli* and *Salmonella* infections usually cause diarrhea and intestinal inflammation and lead to the disorder of the intestinal flora and the destruction of the intestinal barrier. They cross the intestinal barrier into the blood and reach other extraintestinal organs and cause the aggravation of the symptoms of the infection. Some studies have shown that bacteriocins (e.g., microcin and colicin) have an inhibitory effect on *E. coli* and *Salmonella in vitro* and can effectively reduce the numbers of *E. coli* (e.g., O157: H7) and *Salmonella* in the infected mice, improving the adverse effects caused by these pathogens [26–28]. In addition, some bacteriocins (e.g., subtilosin and gassericin E) have a significant inhibitory effect on the pathogens (e.g., *Gardnerella vaginalis*) which can cause the bacterial vaginal diseases [29, 30].

Interestingly, some studies have shown that bacteriocin alone or in combination with antibiotics can not only broaden the antibacterial spectrum (even effective against antibiotic-resistant bacteria) but also significantly reduce the MIC value [20, 31–34]. For example, Singh et al. reported that the combinations of nisin-ceftriaxone and nisin-cefotaxime were found to be highly synergistic against *S. enterica* serovar typhimurium than in those treated with drugs alone, specifically manifested in lower MIC value and less organ cell load [31]. This suggests that bacteriocins could be considered an effective way to reduce the spread of antibiotic resistance.

### 2.2. Inhibiting Virus Infections

Viral infections can attack and destroy the immune system, leading to the formation of malignant tumors. Current treatments for viral infections are mainly chemical drugs, such as inhibitors of DNA polymerase activity that inhibit the replication of the virus [35]. However, the virus is prone to mutate and easily leads to be resistant to these drugs. Therefore, the search for new antiviral drugs is imminent. It has been reported that certain bacteriocins are demonstrated to show antiviral activities to a variety of viruses. Herpes simplex virus types 1 and 2 (HSV-1 and HSV-2) are human viral pathogens that can cause serious clinical conditions including genital ulcerations, corneal blindness, and encephalitis, and over 530 million people worldwide are infected with HSV-2 [36]. Studies reported that several bacteriocins show inhibitory effects against HSV. For example, subtilosin targets intracellular transport of viral glycoproteins in the late stages of the viral replication cycle to exert antiviral or virucidal effects [37]. Similarly, enterocin CRL35 affects the late steps of virus multiplication [38, 39] and labyrinthopeptin A1 targets the glycoproteins, exerting an antiviral effect [40, 41]. In addition, bacteriocins have been reported to have antiviral or virucidal effects against a variety of other viruses, such as human immunodeficiency virus (HIV), zika virus, and dengue virus [37, 42]. Compared to be antibacterial agents, bacteriocins have been much less studies as antiviral agents, and the mechanisms of bacteriocins involved are less well understood and need further research.

### 2.3. Inhibiting Parasite Infection

There are 342 species of helminth parasites and 70 species of protozoan parasites in humans [43]. The relationship between the parasite and the host is complex, as it may either promote host health or cause diseases [44, 45]. Protozoa such as *Plasmodium*, *Trypanosoma*, and *Entamoeba* can cause serious diseases (e.g., malaria, sleeping sickness, and amoebic dysentery) in humans [46, 47]. Several bacteriocins have been reported to have an inhibitory effect on some parasites and can ameliorate diseases caused by parasites. For example, AS-48 is a head-to-tail cyclized peptide, synthesized by *Enterococcus faecalis*. It not only has bactericidal effect on many G^+^ bacteria and several G^−^ bacteria but also effectively reduces the number of *Trypanosoma cruze* by mitochondrial membrane depolarization and reactive oxygen species production, improving the symptoms of Chagas' disease [48]. AdDLP is the first bacterial defensin-like peptide identified in the G^−^ bacterium *Anaeromyxobacter dehalogenans*. 10 *μ*M AdDLP can kill 100% of *Plasmodium falciparum* without harming mammalian red blood cells [49]. Although the research about bacteriocins inhibiting parasites are still limiting, bacteriocins are potential to be an effective drug to fight against parasite infection.

## 3. Anticancer Activities

Cancer is a major public health problem worldwide and is the leading cause of death in the global [50]. Although there have been new breakthroughs in cancer research in recent years, there are still many challenges that need to be addressed, and the prevention and treatment of cancer need to be further explored continuously. Cancer occurs when the cells that line the tissue become abnormal and grow out of control. With the enhancement of migration ability, some cancers might even be present without any signs or symptoms [51, 52]. Inhibiting the proliferation and migration of cancer cells is an effective measure to prevent and treat cancer.

**Figure 2:**
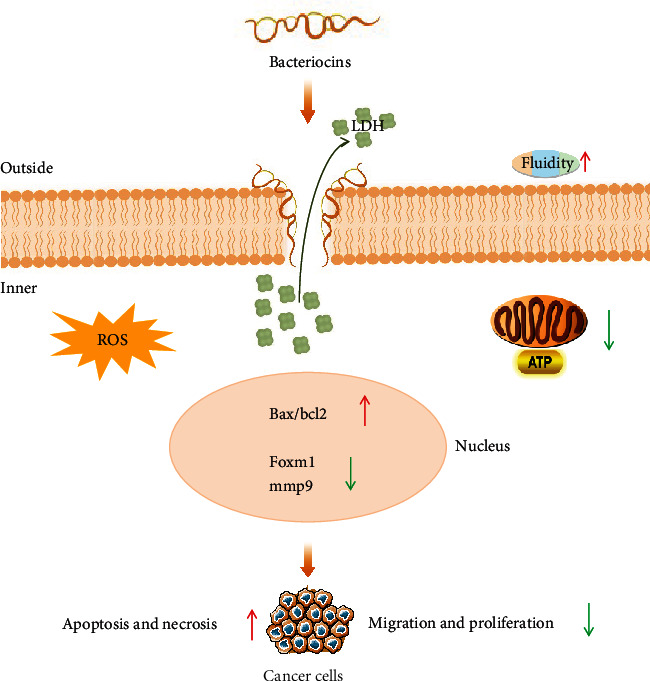
Bacteriocins inhibit the development of cancer by inhibiting the growth of cancer cells through various mechanisms. Bacteriocins increase the fluidity of cell membranes and form ion channels on cancer cell membranes, increasing the release of LDH. Bacteriocins promote the accumulation of intracellular ROS, increase the apoptotic index (bax/bcl2), reduce the expression of FOXM1 and MMP9, inhibit mitochondrial energy metabolism and glycolysis, reduce its energy supply leading to apoptosis and necrosis, or inhibit its migration and proliferation, which ultimately promotes the apoptosis and necrosis, inhibiting the migration and proliferation of cancers.

In recent years, researches on the anticancer effect of peptide have gradually become the focus of attention. Bacteriocins have shown anticancer activities such as killing and inhibiting invasion of some cancer cells.  Table 2 and Figure 2 summarize the anticancer effects of bacteriocins and the mechanisms reported so far, including induction of cell apoptosis, blocking of cell cycle, inhibition of cell migration, and destruction of cell membrane structure.

Nisin can induce the apoptosis of a wide range of cancer cells (e.g., HNSCC, SW480, LS180, HT29, Caco2, SW1088, A375, and IMR-32) [53–59] through multiple mechanisms. After treatment with different concentrations of nisin, the apoptosis index (i.e., bax/bcl-2) of cancer cells was increased, the cell cycle was arrested, and the expression of genes related to proliferation and migration (e.g., *cea*, *ceam6*, and *mmp2f*) were suppressed. In addition, nisin also induces the cell membrane damage, promotes the release of lactate dehydrogenase (LDH), increases the accumulation of reactive oxygen species (ROS), and inhibits the mitochondrial respiration and glycolytic metabolism (lead to cancer cells running out of energy). Interestingly, nisin can also be used in combination with anticancer drugs to significantly enhance their anticancer effects *in vivo*. Preet et al. [60] reported that nisin as an adjunct can promote the effects of doxorubicin against DMBA-induced skin carcinogenesis by improving histopathological features, promote cell apoptosis of tumor, and increase superoxide dismutase (SOD) levels, thereby reducing the average load and volume of the tumor. Similarly, Rana et al. [61] demonstrated that nisin and 5-FU combination be synergistic against DMBA-induced skin cancer and could promote the rapid removal of tumors *in vivo*. These results point towards the possible use of bacteriocins as an adjunct to anticancer drug to prevent local tumor invasion, metastasis, and recurrence and develop alternate strategies to combat currently and developing drug resistance in cancer cells.

Apart from nisin, laterosporulin10 (LS10), a class IId bacteriocin produced by *Brevibacillus laterosporus* SKDU10, not only effectively inhibits pathogens [21] (i.e., *M. tuberculosis* and *S. aureus*) but also kills a variety of cancer cells (e.g., MCF-7, HEK293T, HT1080, HeLa, and H1299 cell lines) at 10 *μ*M by destroying the membrane structure. Interestingly, it shows low toxicity towards normal prostate epithelium cells (RWPE-1) [62]. Microcin E492 produced by *K. pneumoniae* can trigger cancer cells to form ion channels, resulting in cell shrinkage, DNA fragmentation, extracellular exposure of phosphatidylserine, caspase activation, and loss of mitochondrial membrane potential, inhibiting the growth of HeLa, Jurkat RJ 2.25, and Ramos cell lines at the concentration more than 5 *μ*g/mL. Like LS10, microcin E492 also had no effect on normal cells (KG-1 and a primary culture of human tonsil endothelial cell) [63]. Pediocin CP2, a class IIa bacteriocin from *Pediococcus acidilactici* MTCC 5101, can affect cell division and DNA synthesis and induce programmed cell death of multiple cancer cells (MCF-7, HepG2, and HeLa) at 25 *μ*g/mL without selective cytotoxicity. [56, 64] In addition, plantaricin A [65] from *Lactobacillus plantarum*, pediocin PA-1 [56] from *P. acidilactici* K2a2-3, chaxapeptin [66] from *Streptomyces leeuwenhoekii* C58, and duramycin [67] from *Streptoverticillium griseoverticillatum* have been reported to inhibit Jurkat, HeLa, A549, and MCA-RH 7777 cell lines, respectively. Thiostrepton, produced by *Streptomyces*, is an exciting bacteriocin that was reported to have *in vivo* anticancer properties as of nisin. Thiostrepton not only forms a tight complex with the forkhead box M1 (FOXM1, a key regulator of the cell cycle) binding domain and inhibits FOXM1 expression, inhibiting MCF7 cell *in vitro* at 10 *μ*M, but also decreases FOXM1 expression and acts as a proapoptotic agent, thereby inhibiting endometriosis and reducing MMP9 and bcl-2 levels *in vivo* at 150 mg/kg [68–70]. Therefore, many bacteriocins have the potential to be used as antitumor agents by interfering with some aspect of cancer progress. They have a significant potential for developing as antitumor drugs.

## 4. Anti-Inflammation and Immunomodulation Activities

**Figure 3:**
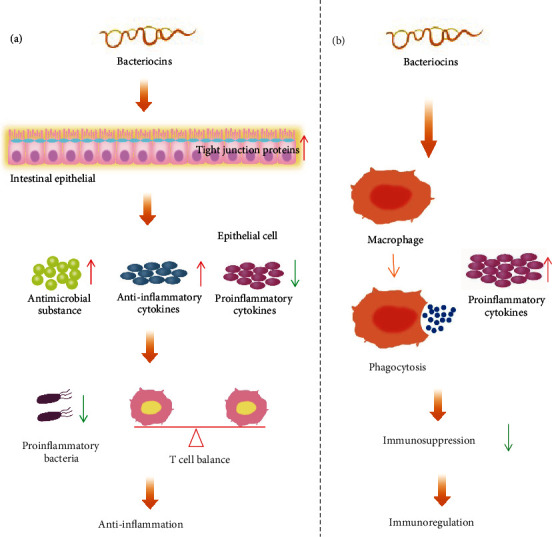
Anti-inflammation and immunomodulation effect of bacteriocins. (a) For the anti-inflammatory effect, some bacteriocins can increase anti-inflammatory cytokine levels, decrease proinflammatory cytokine levels, and maintain the balance between immune cells by inhibiting the activation of inflammatory signaling pathways in a state of inflammation. Some bacteriocins can act directly on pathogenic bacteria or reduce colonization of pathogenic bacteria by stimulating the production of antimicrobial substances. Some bacteriocins can promote the expression of intestinal tight junction proteins and strengthen the intestinal barrier. (b) For immune regulation, some bacteriocins can promote the body to produce inflammatory cytokines and promote the phagocytosis of macrophages, thus boosting the immunity and achieving immune regulation in the immunosuppressive state.

The immune system is a complex network of cells, tissues, and organs that work together to protect the body from harmful substances and defend against disease, which plays an important role in maintaining the health of human [71]. Many diseases are linked to disturbances in the immune system, such as inflammation and immune deficiency [72]. Bacteriocins also have anti-inflammatory and immune-modulatory effects as detailed in Table 3 and Figure 3. Bacteriocins can inhibit the inflammatory effects caused by pathogen-associated molecular patterns (PAMPs) or other irritants by modulating cytokine levels. This is characterized by an increase in anti-inflammatory cytokines and a decrease in proinflammatory cytokines by regulating the activation of certain pathways, such as Toll-like receptor (TLR), nuclear factor kappa-B (NF-*κ*B), and mitogen-activated protein kinase (MAPK) signaling pathways. Bacteriocins also promote the secretion of antimicrobial substances from epithelial cells to kill proinflammatory bacteria. And they inhibit the infection-induced inflammation and migration of pathogens by increasing the expression of tight junction proteins (TJP), strengthening the intestinal barrier, and reducing the invasion of proinflammatory pathogens into the bloodstream and extraintestinal organs.

Nisin has been reported to have a significant anti-inflammatory effect *in vitro* and *in vivo*. Nisin A can increase the activity of human keratinocytes HaCaT, inhibit LPS-induced proinflammatory cytokine levels (TNF-*α*), and reduce bacterial growth, promoting wound healing [73]. Nisin Z inhibits *S. agalactiae* and *S. aureus* and leads to a significantly decreased milk somatic cell count in cows with mastitis, thus effectively relieving the symptoms of mastitis [74]. Nisin P from *Streptococcus lactis* SMN003 reduces uterine inflammation in rats by regulating the concentration of proinflammatory and anti-inflammatory cytokines (regulate the levels of B7-2, IFN-*γ*, IL-2, and IL-8) and normalized uterine neutrophils thus restoring endometrial architecture [75].

Plantaricin EF, class IIb bacteriocins which are produced by *L. plantarum*, can promote the expression of TJP in obese mice, increase the integrity of intestinal barrier, reduce the weight of obese mice, and reduce the inflammation of fat [76]. Microcin M produced by *E. coli* MC4100 mediates the competition of *Enterobacter* in inflammatory bowel, reduces the colonization of intestinal pathogenic bacteria, and reduces intestinal inflammation [77]. A lasso peptide of microcin J25 from *E. coli* can reduce the levels of IL-6, IL-8, and TNF-*α* to prevent intestinal damage and inflammation caused by ETEC K88. Microcin J25 also can effectively improve the production performance of *salmonella*-infected broilers, systemic inflammation, and the composition of fecal microflora [32, 78–80]. This is inconsistent with the commonly held view that bacteriocins have little effect on the structure of intestinal flora. It might be due to the special structure of microcin J25 (a lasso peptide), which makes it insensitive to proteases and thus affects intestinal microorganisms. Besides, microcin J25 also improves the fecal microbiota of weaned piglets, thereby promoting piglet growth, apparent total digestibility, and intestinal barrier function [32]. Salivaricin LHM from *Lactobacillus salivarius* inhibits the growth and biofilm formation of *Pseudomonas aeruginosa* (often cause nosocomial infection) and can also reduce the inflammation and prevent injury caused by *P. aeruginosa* infection. So, the salivaricin LHM has anti-inflammation effect *in vivo* and *in vitro* [81].

In fact, whether it is an anti-infective, antitumor, or anti-inflammatory effect, this is inseparable from immune regulation. Nisin can not only reduce the level of proinflammatory factors to play an anti-inflammatory function but also promote the secretion of proinflammatory factors under certain conditions. For example, nanoparticles synthesized by nisin and Ag (nisin-Ag) increased the level of the proinflammatory cytokine IL-12 in macrophages [82]. Interestingly, nisin promotes the proliferation of peripheral blood mononuclear cells (PBMC), stimulate the production of IL-1 and IL-6, and increase the proportion of CD4^+^ CD8^+^ T cells. Contrary, when PBMC is stimulated by LPS, nisin reduces the production of LPS-induced proinflammatory cytokine IL-6 [83]. It indicates that nisin has strong immune-modulatory activity. Sublancin (1.0 mg/kg) can enhance macrophage function, increase CD 4^+^ and CD 8^+^ cells, and protect mice from MRSA infection [84]. It also prevents cyclophosphamide-induced immunosuppression in mice and inhibits NF-*κ*B activation to balance the immune response during infection, alleviating intestinal inflammation [85]. Thiostrepton is a kind of thiopeptide, which can inhibit the psoriatic inflammation, which induced by TLR7, TLR8, and TLR9 *in vivo* [86].

As mentioned above, bacteriocins have a wide range of biological activities, suggesting that they may be used as anti-infective compounds and effective therapeutic agents in the treatment of a number of immune-related diseases, and they may even have promising applications in cancer therapy.

## 5. Opportunities of the Application of Bacteriocins in Human Health

### 5.1. Delivery Systems for Bacteriocins

Bacteriocins are an essential class of polypeptide substance. They are reported to be involved in improving gut health, such as reducing pathogenic bacteria colonization, improving the intestinal barrier, and alleviating intestinal inflammation. Besides, bacteriocins are not easy to cause drug resistance and have little influence on commensal flora. For example, thuricin CD, a posttranslationally modified bacteriocin produced by *B. thuringiensis* DPC 6431with an activity against *C. difficile*, has potential as a targeted therapy in the treatment of *C. difficile*-associated infection while also reducing collateral impact on the commensal flora [87]. Some bacteriocins, such as lasso peptide microcin J25, have stable s structures to avoid degradation by proteases in digestive tract [80]; however, most bacteriocins are susceptible to be degraded by proteases when administered orally, leading to the loss of antimicrobial activity. As a result, only a small fraction of bacteriocins has been tested *in vivo* by intraperitoneal injection, nasal feeding, or applying to skin. Therefore, effective delivery methods are necessary to ensure that they are not degraded when they reach the intestine.

Nanoparticles (i.e., metal nanoparticles, organic nanoparticles, nanospheres, and nanofibers), probiotics, and gels may be used as bacteriocin delivery systems [88]. For example, nisin nanoparticles have sustained release effect compared with nisin alone, prolonging the action time for the recurrent vaginal candidiasis treatment [89], and slow release contributes to prolonging the duration of the effect. In addition, some delivery modes enhance the activity of bacteriocins. For example, compared with enterocin alone, enterocin-capped silver nanoparticles (En-SNPs) synthesized by enterocin and nanosilver have stronger antibacterial activity against multiple foodborne pathogens (i.e., *E.coli* ATCC 25922, *B. cereus*, *K. pneumoniae*, *L. monocytogenes*, *M. luteus*, *P. acidilactici* LB42, *S. flexneri*, and *S. aureus*) [90]. Mohid et al. described five bacteriocins which are effective against *M. tuberculosis*. After being embedded in liposomes (phosphatidylcholine: cardiolipin =3 : 1), four of them are better than rifampicin (traditionally used to treat *M. tuberculosis* infection) *in vivo* [91]. However, as the best of our knowledge, those delivery systems has only little effect to solve the protease degradation problem.

Many probiotics have been reported to tolerate the gastrointestinal environment and successfully colonize the intestine. Consequently, bacteriocin-producing probiotics act as vehicles to transport the bacteriocins to the intestinal tract for their beneficial effects. Malvisi et al. found that nisin-producing strains show stronger antimicrobial activity against mastitis-causing bacteria than nonnisin-producing strains [92]. Similarly, Yin et al. demonstrated greater anti-inflammatory activity in mice fed *L. plantarum* compared to the mutant strain lacking the bacteriocin plantaricin [93]. In turn, the production of bacteriocins promotes the colonization of probiotic bacteria, facilitating their occupation of ecological niches and reducing the colonization of pathogenic bacteria [94]. Thus, bacteriocin-producing strains can be used as vehicles to help bacteriocins colonize and function in gastrointestinal research.

### 5.2. Increasing Bacteriocins Production and Activity by Genetic Engineering

The production of bacteriocins in the original strains is usually low, and some bacteriocins are encoded by plasmids and are not produced in stable yields. Increasing the yield of bacteriocins is of great importance for the research and application of bacteriocins. In addition, the activity of some bacteriocins has to be improved in practice, which can also reduce the amount of bacteriocins used and thus indirectly solve the problem of insufficient bacteriocin production. Genetic engineering is a good solution to both of these problems. For the increase of bacteriocin production, Ni et al. used the shuttle expression vector pMG36e with the strong constitutive promoter p32 to further enhance the production of nisin by overexpressing the genes *nisA*, *nisRK* and *nisFEG* in *L. lactis* LS01 [95]. Kong et al. obtained the 14.5 kb complete gene cluster of nisin from *L. lactis* K29 nisin-producing bacteria, transferred it into *L. lactis* MG1363 with pCCAM*β*1 plasmid, and overexpressed the core peptide gene *nisA*, thereby increasing the yield of nisin [96]. For the enhancement of the bacteriocins activity, Zhou et al. attached the tail (PRPPHPRL) of apidaecin 1b to nisin, and the activity of nisin against *E. coli* CECT101 was increased by more than twofold [97]. Recently, Steven et al. have improved the activity of antimicrobial peptides against pathogenic bacteria and broadened the spectrum of inhibition by combinatorically shuffling the peptide modules of 12 lanthipeptides. [98] Overall, genetic engineering is an effective approach to increase bacteriocin production and enhance bacteriocin activity.

### 5.3. Bacteriocins as Narrow-Spectrum Antimicrobials to Be Needed for Healthy Human Microbiota

The human microbiota is composed of a diverse community of bacteria, and the microbial composition and abundance changes are related to a range of human diseases. Broad-spectrum antibiotic administration could dramatically reduce gut microbiota diversity and cause many side effects. For example, antibiotic-associated diarrhea occurs when the balance of “good and bad bacteria” in the gastrointestinal is disrupted after taking antibiotics.

Many bacteriocins have a relatively narrower spectrum and targeted against a little specific bacteria compared to antibiotics which have a broad-spectrum activity. As bacteriocins usually inhibit closely related bacteria, some bacteriocins produced by pathogens showed specific antimicrobial activity to the related pathogenic bacteria. For example, lantibiotic suicin from *S. suis* has an inhibitory effect against *S. gordonii* which can cause human sepsis [99]. Klebocin from clinical isolates of *K. pneumonia* show antimicrobial activity to pathogenic species from enterobacteriaceae [100]. Aureocins produced by *S. aureus* has a strong inhibitory effect on *S. aureus* and *S. agalactiae* [101]. In addition, bacteriocins have no impact on normal microbial flora due to their narrow spectrum. For instance, diffocin is produced by *C. difficile* CD4 and can specifically kill other *C. difficile* strains. The modified diffocins completely prevented the intestinal settlement of *C. difficile* without infecting gut flora by oral administration in mice [102]. Similarity, thuricin CD produced by *B. thuringiensis* DPC 6431 showed elimination of *C. difficile* and has little impact on normal genera in gut [87]. Microcin J25 intervention in a diarrhea model reduces pathogenic *E. coli* colonization while improving intestinal microbiology [32]. Therefore, bacteriocins have a great potential to be used as a narrow-spectrum bacterial inhibitor for the treatment of infection-related diseases in human.

In practice, the safety of some bacteriocins is of concern as their producing bacteria are pathogenic. Therefore, for these bacteriocins, using purified bacteriocins or heterogenous probiotic bacteria expressing the bacteriocin rather than the producing strains is applicable. It is worth mentioning that a rigorous safety assessment of bacteriocins *in vitro* and *in vivo* is necessary before practical application, regardless of whether the source is probiotic or pathogenic.

## 6. Conclusion and Prospect

This review highlights the potential of bacteriocins as novel therapeutic treatments in microbe infection, cancer, and immune system in human body. There is an abundance of knowledge on the bacteriocins applied in food industry, agriculture, and veterinary fields. However, there is limiting available *in vitro* and *in vivo* data regarding human health. Due to the sensitivity of some bacteriocins to protease, many studies on the activity of bacteriocins are confined to *in vitro* experiments and have not been deeply studied in the model of animals. Some posttranslationally modified bacteriocins show higher stabilities in the digestive tract, while less is known about their impact in an in *vivo* environment. The bacteriocin delivery system might be an important path to solve the degradation of bacteriocin in the digestive tract. Besides, more and more bacteriocin biosynthesis clusters are predicted using bioinformatic approaches; however, the bacteriocin-producing strain is not easy to obtain. The combination of high-throughput sequencing and culture omics may provide ideas for the discovery of new bacteriocins and their producing strains. More research related to the cytotoxicity, hemolytic activity, distribution, and metabolism of bacteriocins is needed to explore their contribution to human health. The unique antibacterial mechanism of bacteriocins compared to conventional antibiotics makes them a potential alternative to antibiotics. Further studies on the function and mechanism of action of bacteriocins will help advance their practical application in anti-infection, anticancer, and anti-inflammation or immunomodulation.

## Figures and Tables

**Table 1:** Antimicrobial effects of bacteriocins.

Bacteriocins -classification	Producing bacteria	Target organism	Mode of action	Model	Security
Nisin lanthipeptide [17, 103–105]	*L*. *lactis*	*S. aureus*, *C. difficile*	Lipid II binding and pore formation	*In vitro*, mice and rats (intraperitoneal injection and nasal administration)	FDA approved and generally regarded as safe
NAI-107 lanthipeptide [106, 107]	*Microbispora* sp.	*S. aureus*	Inhibits the synthesis of peptidoglycan	*In vitro* and mice (intravenous and subcutaneous administration)	Low acute toxicity
Mutacin B-Ny266 lanthipeptide [108, 109]	*S. mutans*	*S. aureus*, *Neisseria Helicobacter*	Unknown	*In vitro* and mice (intraperitoneal injection)	Not evaluated
OG716 lanthipeptide [110]	*S. mutans* JH1140	*C. difficile*	Binding the pyrophosphate moiety of lipid-II	*In vitro* and hamster	Low toxicity
Mersacidin lanthipeptide [22, 111–113]	*Bacillus* sp. HIL-Y85/54728	MRSA	Inhibits bacterial cell wall biosynthesis by complexing lipid II	*In vitro* and mice (nasal administration and subcutaneously administered)	Not evaluated
Actagardine A lanthipeptide [114]	*A. garbadinensis* ATCC 31049	*C. difficile*, VRE MRSA	Inhibits cell wall biosynthesis by binding to lipid II and blocking transglycosylation	*In vitro*	Not evaluated
NVB302 lanthipeptide [115, 116]	Derivative of deoxyactagardine B from *A. liguriae*	*C. difficile*	Binding to lipid II	*In vitro* and hamsters (oral gavage) and *ex vivo* gut model	Nontoxic
NVB333 lanthipeptide [117]		*S.aureus*		*In vitro* and mice (i.v. injection)	No signs of any drug-related adverse effects
Lacticin 3147 lanthipeptide [118]	*L. lactis* DPC3147	*C. difficile*, *L. monocytogenes*	Binding to lipid II and lytic	*In vitro*	Not evaluated
Lassomycin class I-lasso peptide [119]	*Lentzea kentuckyensis*	*M. tuberculosis*	Target the ATP-dependent protease	*In vitro*	Not evaluated
Microcin J25 lasso peptide [26, 80, 120, 121]	*E. coli*	*Salmonella, E. coli*	Inhibiting RNA polymerase and increasing superoxide production	*In vitro* and mice	No cytotoxicity
Enterocin AS-48 head-to-tail cyclized peptides [122–125]	*E. faecalis*	*M. tuberculosis*	Accumulating a positive charge on the membrane surface and disrupts the membrane potential	*In vitro* and macrophages	No cytotoxicity
Thiostrepton thiopeptide [126, 127]	*Streptomyces* sp.	*M. abscessus*	Binding to a site on 23S rRNA and inhibits elongation factor-dependent reactions	*In vitro*, zebrafish and macrophages	US FDA-approved drug
Durancin 61A glycosylated bacteriocin [128, 129]	*E. durans* 61A	*C. difficile*, VRE, MRSA, *L. innocua*	Targeting the bacterial membrane	*In vitro*	Not hemolytic
Thuricin CD sactipeptide [87, 130, 131]	*B. thuringiensis* DPC 6431	*C. difficile*, *L. monocytogenes*	Permeabilize and depolarize the membrane	*In vitro* and mice	Not evaluated
Ruminococcin C sactipeptide [132, 133]	*R. gnavus* E1	Pathogenic clostridia and MDR strains	Inhibiting nucleic acid synthesis in a metronidazole-like manner	*In vitro*	Not toxic to eukaryotic cells
Gassericin E head-to-tail cyclized peptides [30]	*L. gasseri* EV1461	Multiple pathogens associated with bacterial vaginosis	Unknow	*In vitro*	Not evaluated
Microcin H47 [134]	*E. coli* Nissle 1917	*E. coli*	Targeting the F_0_ proton channel of ATP synthase	*In vitro*	Not evaluated
Microcin E492 [135]	*K. pneumoniae* RYC492	*K. Enterobacter E. coli Salmonella* sp.	Permeabilize the inner membrane with the mannose permease	*In vitro*	Induces apoptosis in some human cell lines
Microcin M [136]	*E. coli* Nissle 1917	*E. coli Salmonella* sp.	Compete against other enterobacteria that utilize catecholate siderophores	*In vitro*	Not evaluated
Lactocin 160 [137, 138]	*L. Rhamnosus*	*G. Vaginalis* *Bacillus pertussis*	Causing the efflux of ATP molecules and dissipative the proton motive force	In epivaginal	Minimal irritation
Enterocin CRL35 class IIa [25, 139]	*E. mundtii*	*L. monocytogenes*	Forming holes in the cell wall and cell membrane	*In vitro* and mice (orally administrated)	Not evaluated
Lactocin AL705 class IIa [140, 141]	*L. curvatus*	*L. monocytogenes*	Disrupting quorum sensing through a signal molecule inactivation	*In vitro*	Not evaluated
Pediocin PA-1 class IIa [24, 142, 143]	*P. acidilactici*	*L. monocytogenes*	Forms hydrophilic pores in the cytoplasmic membrane	In intra-gastric administration	Commercial applications with no adverse effect
Laterosporulin10 class IId [21]	*B. laterosporus* SKDU10	*S. aureus*, *M. smegmatis*	Membrane permeabilization	*In vitro* and macrophages	No hemolytic activity
Subtilosin class II [29, 144, 145]	*B. subtilis*	*G. vaginali*, *L. monocytogenes*, *S. agalactiae*	Binding to lipid bilayers, results in membrane permeabilization	In epivaginal	Human cells remained viable after prolonged exposures to subtilosin
Colicin Z class III [27]	*E. coli* B1356	*E. coli Shigella*	Via cjrc receptor recognition and cjrb- and exbb- and exbd-mediated colicin translocation	*In vitro*	Not evaluated
Colicin F Y class III [28, 146]	*E. coli*	*Y. enterocolitica*	Yiur-mediated reception, tonb import, and cell membrane pore formation	In mice	Not evaluated
Diffocin class III [102, 147]	*C. difficile CD4*	*C. difficile*	Dissipating the membrane potential	In vitro and mice	Not evaluated
ESL5 [148]	*E. faecalis* SL-5	*P. acnes*	Unknown	*In vitro* and human	Not evaluated
Bacteriocin OR-7 [149]	*L. salivarius* NRRL B-30514	*C. jejuni*	Unknown	In chicken	Not evaluated
Bacteriocin E 50-52 class IIa [150]	*E. faecium* NRRL B-30746	*S. enteritidis*	Unknown	In chicken	Not evaluated
Subtilosin class II [37]	*B. subtilis*	HSV-1 and HSV-2	Inhibiting virus multiplication	*In vitro*	Human cells remained viable after prolonged exposures to subtilosin
Labyrinthopeptin A1 lanthipeptide [40, 41, 151]	*A. namibiensis* DSM 6313	HSV, HIV, zika virus, and dengue virus	Acting as an entry inhibitor possibly by targeting the HSV glycoproteins	*In vitro*	Does not harm the vaginal epithelium or the normal vaginal lactic acid flora
Enterocin CRL35 class IIa [38, 39]	*E. mundtii*	HSV-1 and HSV-2	Affecting a late step of virus multiplication	*In vitro*	Low cytotoxicity for eukaryotic cells
Mundticin ST4SA class IIa [42]	*E. mundtii* ST4V	HSV-1, HSV-2, poliovirus and measles virus	Unknown	*In vitro*	Not evaluated
Enterocin AS-48 class I-head-to-tail cyclized peptides [48, 125, 152]	*E. faecalis*	*Trypanosoma cruzi*	Mitochondrial membrane depolarization and reactive oxygen species production	*In vitro* and mice	No cytotoxicity
Addlp class II [49]	*A. dehalogenans*	*Plasmodium falciparum*	Unknown	*In vitro*	Nontoxic to mammalian cells

**Table 2:** Anticancer effects of bacteriocins.

Bacteriocins	Classification	Source	Target cancer cells (mechanism) or effects *in vivo*
Nisin	Lanthipeptide	*L. lactis*	SW1088 [57]; HNSCC (arresting the cell cycles) [53]; SW480 (increasing the apoptosis index of bax/bcl-2) [54]; LS180, HT29, and Caco2 (decreasing the expression of genes related to proliferation and migration) [55]. IMR-32 (enhancing cell membrane fluidity) [59]. Combining with doxorubicin can reduce the tumor volume of skin cancer in mice [60]. Decreasing the IC50 of 5-FU on A431 cells and promote the elimination of tumors in mice [61, 153]
Nisin Z	Lanthipeptide	*L. lactis*	A375 (inducing cell membrane damage, increasing ROS accumulation, inhibiting mitochondrial respiration and glycolytic metabolism) [58]; HNSCC (induces apoptosis and reduces proliferation and clone formation). Reduces the occurrence of tumors in mice and prolongs survival [154]
Bovicin HC5	Lanthipeptide	*S. bovis* HC5	MCF-7 and HepG2 [155]
Duramycin	Lanthipeptide	*S. griseoverticillatum*	MCA-RH 7777 (enhancing the sensitization) [67]
Chaxapeptin	Lasso peptide	*S. leeuwenhoekii* C58	A549 [66]
Thiostrepton	Thiopeptide	*S. aureus*	MCF-7 (inhibiting FOXM1expression) [68, 69]. Inhibiting endometriosis lesions and reducing the levels of MMP9 and bcl-2 in rats [70]
Microcin E492	Microcin	*K. pneumoniae*	HeLa, Jurkat, and Ramos (forming ion channels) [63]. Tumor inhibition in SW480 and SW620 zebrafish xenograft models [156]
Pediocin CP2	Class IIa	*P. acidacticactic* CP2	MCF-7, HepG2, Sp2/0-Ag14 and HeLa (affecting cell division and DNA synthesis) [64]
Pediocin PA-1	Class IIa	*P. acidilactici* K2a2-3	HT29 and HeLa [24]
Plantaricin A	Class IId	*L. Plantarum*	Jurka (disrupting cell membrane structure) [65]
Laterosporulin 10	Class IId	*B. laterosporus* SKDU10	MCF-7, HEK293T, HT1080, HeLa and H1299 (disrupting cell membrane structure) [62]

**Table 3:** Anti-inflammation and immunomodulation effects of bacteriocins.

Bacteriocins	Classification	Resource	Highlights
Nisin A	Lanthipeptide	*L. lactis*	Decreasing the levels of IL-6, IL-8, and TNF-*α* and reduce the growth of bacteria in the wound [73]
Nisin Z	Lanthipeptide	*L. lactis*	Inhibiting *S. agalactiae* and *S. aureus*, alleviating mastitis in cows [74]
Nisin	Lanthipeptide	*L. lactis*	Increasing the level of IL-12 in macrophages [82], adjust the levels of inflammatory factors in both directions and promote immune balance [83]. Decrease the levels of TNF-*α*, TNF-*β*, NF-*κ*B, IL-1, and ROS in mice [153]
Nisin P	Lanthipeptide	*S. lactis* SMN003	Regulating cytokine concentration to reduce uterine inflammation in rats [75]
Thiostrepton	Thiopeptide	*Streptomyces* sp.	Inhibiting psoriasis-like inflammation induced by TLR7, TLR8, and TLR9 [86]
Microcin M	Microcin	*E. coli* MC4100	Inhibiting intestinal pathogenic bacteria and reducing intestinal inflammation [77]
Microcin J25	Lasso peptide	*E. coli*	Improving intestinal inflammation of broiler and mouse caused by *Salmonella* and ETEC [78, 79]
Sublancin	Glycocin	*B. subtilis* 800	Enhancing macrophage function, increase CD 4^+^ and CD 8^+^ cells, thereby enhancing immune response [84, 85]. Inhibiting NF-*κ*B, relieving intestinal inflammation [157]
Gassericin A	Circular bacteriocins	*L. gasseri* LA39	Binding to KRT19 thus promote fluid absorption and decrease secretion early-weaned piglets [158]
Salivaricin LHM	Class II	*L. salivarius*	Inhibiting inflammation caused by *P. aeruginosa*, with immune regulation in mice [81]
Plantaricin EF	Class IIb	*L. plantarum*	Reducing obesity and fat inflammation [76]
Lmo2776	Class IId	*L. monocytogenes*	Targeting the commensal *P. copri* and modulate intestinal infection in mice [159]
